# Excellent Thermally Conducting Ni Plating Graphite Nanoplatelets/Poly(phenylene sulfone) Composites for High-Performance Electromagnetic Interference Shielding Effectiveness

**DOI:** 10.3390/polym13203493

**Published:** 2021-10-12

**Authors:** Zhang Chen, Ting Yang, Lin Cheng, Jianxin Mu

**Affiliations:** 1 Shenzhen WOTE Advanced Materials Co., Ltd., Shenzhen 518000, China; chenzhang1983823@126.com; 2 Key Laboratory of High Performance Plastics, Ministry of Education, National & Local Joint Engineering Laboratory for Synthesis Technology of High Performance Polymer, College of Chemistry, Jilin University, Changchun 130012, China; yangting1988@jlu.edu.cn; 3 Jilin University Special Plastics Co., Ltd., Changchun 130000, China; chenglin21@mails.jlu.edu.cn

**Keywords:** graphene, thermal properties, composites

## Abstract

First, nickel particles were deposited on the surface of graphite nanoplatelets to fabricate highly conductive GnPs@Ni core-shell structure hybrid fillers via electroplating. The modified GnPs were blended with polyphenylene sulfone via the solution blending method, followed by the hot-pressing method to achieve high thermally conducting GnPs@Ni/PPSU composites for high performance electromagnetic interference effectiveness. The results showed that in-plane and through-plane thermal conductivity of the composite at the 40 wt% filler loading could reach 2.6 Wm^−1^K^−1^ and 3.7 Wm^−1^K^−1^, respectively, which were 9.4 and 20 times higher than that of pure PPSU resin. The orientation degree of fillers was discussed by XRD and SEM. Then, heat conduction data were fitted and analyzed by the Agari model, and the heat conduction mechanism was further explored. The testing results also demonstrated that the material exhibited good conductivity, electromagnetic shielding effectiveness and superior thermal stability. Overall, the GnPs@Ni/PPSU composites had high thermal conductivity and were effective electromagnetic shielding materials at high temperatures.

## 1. Introduction

With the advent of the fifth-generation mobile communication and artificial intelligence technology, electronic devices are stepping towards miniaturization and high integration, which makes the power consumption of electronic products increase and the heat dissipation difficult, seriously limiting the development of the next generation of novel electronic components [1,2,3]. Meanwhile, the radiation pollution of electromagnetic wave is becoming increasingly prominent, which leads to the decrease of the service life of sensitive electronic components and endangers human health [4]. Traditional thermal conductive materials (TC) and electromagnetic interference (EMI) materials, such as aluminum foil and copper foil, cannot meet the satisfaction of modern communication, intelligent electronic equipment, automobile and consumer electronics, due to their high density and poor corrosion resistance. Recently, incorporating thermal conductive fillers, such as metal powders [5], carbon nanotubes [6], graphite [7], graphene [8] and boron nitride [9] into polymer matrix to fabricate high λ polymeric composites are widely used in the field of industry, microelectronics, energy, aerospace and so on due to their light weight, chemical corrosion resistance, impact resistance and easy processing [10,11,12].

Metal powders, such as, Ag, Cu, and Ni, with excellent electrical and thermal conductivity, are widely applied in the preparation of high heat conduction and electromagnetic shielding materials. Zhou et al. [13] incorporated Al particles in poly(vinylidene fluoride) (PVDF) matrix by the melt blending method. The λ value of the Al/PVDF composite with 60 vol% Al reached approximately 1.74 Wm^−1^K^−1^, nearly seven times that of the original PVDF matrix. Rivière et al. [14] prepared a series of thermally conductive AgNP/PEEK nanocomposites, and the obtained λ value of the AgNP/PEEK nanocomposite with 8 vol% Ag was significantly enhanced to 0.49 Wm^−1^K^−1^. However, it is still a great challenge to fabricate ideal thermal conductive materials at lower filler loading. Moreover, it has high density and high cost. Recently, researchers have prepared core-shell particles, such as Ag-plated graphite nanosheets and nickel-plated carbon fiber. Hybrid particles with core-shell structure have advantage over traditional carbon materials and metal powder [15,16,17]. For example, Li et al. [18] successfully fabricated Ni-CF/PEEK composites, of which thermal and electrical conductivity can reach up to 1.9 × 10^4^ S/m and 0.65 Wm^−1^K^−1^, respectively. In addition, Ni-plated graphite can improve the interfacial compatibility between the fillers and polymer matrix, further enhancing the bonding strength. Additionally, the developed material not only represents both excellent electrical and thermal conductivity, but is also lightweight and low cost [19]. Hence, Ni-plated graphite is widely used in manufacturing conductive composites. Electroless plating, a new metal surface treatment technology, is widely drawing the attention of scientific researchers because of its simple technology, and energy saving and environmental protection characteristics. Therefore, the electroplating has a wide range of applications in protection performance, decoration, and other functions. The uniform metal plating layer could improve the corrosion resistance and service life of the product.

Graphite nanoplatelets are two-dimensional fillers with high aspect ratio, which are composed of graphene flakes with different stacking layers. GnPs have elicited substantial applications in the field of efficient heat management materials because of its extreme in-plane λ (approximately 3000 Wm^−1^K^−1^) and low interfacial thermal resistance [20,21]. Liu and co-workers [22] reported the epoxy composites filled with particular structure cigarette filter-templated graphene. The perpendicular λ value of the epoxy composites was enhanced to 1.2 Wm^−1^K^−1^, four times higher than that of in-plane λ value (0.298 Wm^−1^K^−1^). However, weak interactions between pure GnPs and polymer matrix as well as intensive Van der Waals forces between graphite nanoplatelets make it prone to aggregation, leading to poor interfacial interactions and, consequently, high thermal resistance [21,23]. In order to solve the mentioned problems, treating by the surface modification, including covalent and non-covalent method, is usually carried out [24]. Improving the interaction of GnPs with polymer matrix without sacrificing the near-perfect structure is still a challenging issue to fabricate high λ composites.

In light of the findings advanced by our previous research team members [18], the surface modification of nano graphite sheet is carried out by chemical plating technology, and nickel nano particles are deposited on the surface of graphite nanoplatelets to prepare the electrical and thermal conductive fillers GnPs@Ni with core-shell structure. The above hybrid fillers were then mixed with polyphenylene sulfone solution, followed by hot-pressing to prepare GnPs@Ni/PPSU composites. The chemical composition and microstructure of GnPs@Ni and its composites samples with different filler loading were characterized. The thermal conductivity, the orientation of layered fillers, and the effects of Ni were investigated. Additionally, EMI shielding, EC value and thermal stability were analyzed.

We hope that by adding high thermal conductivity filler, the polymer will have good thermal conductivity and electromagnetic shielding performance, so as to expand the application of polymers in the field of electronic communication and promote the realization of the goal of replacing steel with plastic.

## 2. Materials and Fabrication

### 2.1. Materials

The PPSU (powder, 800 mesh) of density 1.25 g cm^−3^ was purchased from Jilin University Special Plastics Co., Ltd. (Changchun, China). The GnPs (powder, 99.5%) of density 0.6 g cm^−3^ was purchased from Chengdu Organic Chemicals Co., Ltd. (Chengdu, China). Trisodium citrate dihydrate (C_6_H_5_Na_3_O_7_·2H_2_O, AR), sodium hypophosphite (NaH_2_PO_2_·H_2_O, AR) and *N*,*N*-Dimethylformamide (DMF, AR 99.5%) were obtained from Macklin Biochemical Technology Co., Ltd. (Shanghai, China). Hydrochloric acid (HCl, 37%) and nickel sulfate (NiSO_4_·6H_2_O, AR) were received from Tianjin Chemical Reagent Co., Ltd. (Tianjin, China). The Stannous chloride dihydrate (SnCl_2_, AR) and Palladium chloride (PdCl_2_, AR) was purchased from Aladdin Biochemical Technology Co., Ltd. (Shanghai, China).

### 2.2. Preparation of GnPs@Ni Hybrids

Herein, electroless nickel plating was utilized to prepare GnPs@Ni functional particles with core-shell structure. First, the graphite nanoflakes were extracted with acetone to remove the surface impurities, then filtered and dried at 100 °C in the vacuum oven. The sensitizing solution was prepared with 25 g SnCl_2_, 10 mL concentrated HCl and 1 L H_2_O. An amount of 1 g GnPs were added into the above solution, treated by ultrasonication for 30 min and filtered. Subsequently, the sensitized GnPs were added into the activation solution that was composed of 0.05 g PdCI_2_, 0.01 mL concentrated HCl and 100 mL H_2_O with ultrasonication for 30 min, followed by filtering and washing. The composition of electroless plating bath was 20 g NiCl_2_·6H_2_O, 30 g C_6_H_5_Na_3_O_7_·2H_2_O, 40 g NaH_2_PO_2_·H_2_O and 1 L H_2_O, adjusting pH value in the range of 4.9. The pretreated GnPs were dispersed into the electroplating liquid via ultrasonication at 88 °C. The reaction time was 2 h. Next, the mixture was separated by vacuum assisted filtration through PTFE membrane and washed repeatedly until the filtrate became colorless. The black powders with slight metallic luster (GnPs@Ni) were obtained.

### 2.3. Preparation of GnPs@Ni/PPSU Composites

**Figure 1:**
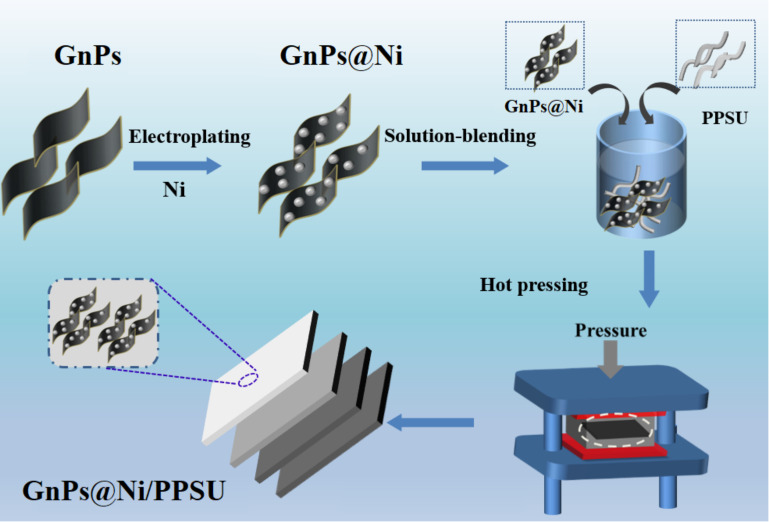
The corresponding schematic diagram of the fabrication for the GnPs@Ni/PPSU nanocomposites.

The prepared Gnps@Ni was added to the beaker containing DMF, and the dispersion of Gnps@Ni was prepared with a concentration of 5 g/L, then treated by ultrasonication for 2 h. A certain amount of PPSU was added to the fully dispersed solution subsequently to prepare a solution containing Gnps@Ni 10 wt%, 20 wt%, 30 wt%, and 40 wt%, respectively. The above solution was stirred at room temperature for 12 h. Then, the mixed solution was poured into a large amount of cold deionized water under stirring. After stirring for 6 h, the solution was filtered and washed with a large amount of deionized water. The obtained filter cake was cut into small pieces and dried in a vacuum oven to constant weight. GnPs@Ni/PPSU composites were beaten into powder with a high-speed mixer and hot-pressed at 280 °C and 30 Mpa. Figure 1 presents the corresponding schematic diagram of the fabrication for the GnPs@Ni/PPSU nanocomposites.

### 2.4. Characterization

The equation used to calculate the thermal conductivity of materials is shown as follows:

*λ* = *α* · *C_p_* · *ρ* (1)

The *ρ*, *α* and *C_p_* in the formula represent density, thermal diffusion (mm^2^s^−1^), and specific heat capacity, respectively. The composite sheet obtained by hot-pressing was made into a small disc with a thickness of 0.8 mm and a diameter of 12.5 mm, then thermal diffusion coefficient of the material at 20 °C was measured by laser flasher (LFA 467 was made by Netzsch in Germany). DSC 821e (Made by Mettler Toledo in Switzerland) was used to measure the heat capacity of the material. The density was measured by a hydrometer. Network analyzer (N5244A PNA-X was made by Agilent in America) was used to measure the electromagnetic shielding effectiveness. Keithley2450 was used to determine the electrical conductivity of the material at 25 °C. We used DIL 806 (Made by TA instruments in America) to measure the thermal expansion coefficient of the material at temperature range from 30 to 180 °C, and the heating rate was set to 5 °C min^−1^. We carried out thermogravimetric analysis of the sample with TGA/DSC1 in nitrogen atmosphere; the temperature range was 100 to 800 °C, and the heating rate is 10 °C min^−1^. The chemical composition of the material was determined by XRD (XRD-6000 was made by Shimadzu Corporation in Japan) and X-ray photoelectron spectroscopy (RSCAKAB250X was made by ThermoVG America). The Micromorphology of the materials was captured by transmission electron microscopy (TEM, Jeol2100 was made by Jeol in Japan) and scanning electron microscopy (SEM, NOVA NANOSEM 450 was made by FEI corporation in America).

## 3. Results and Discussion

### 3.1. Chemical Composition of GnPs@Ni-MWCNTs Hybrid Filler

**Figure 2:**
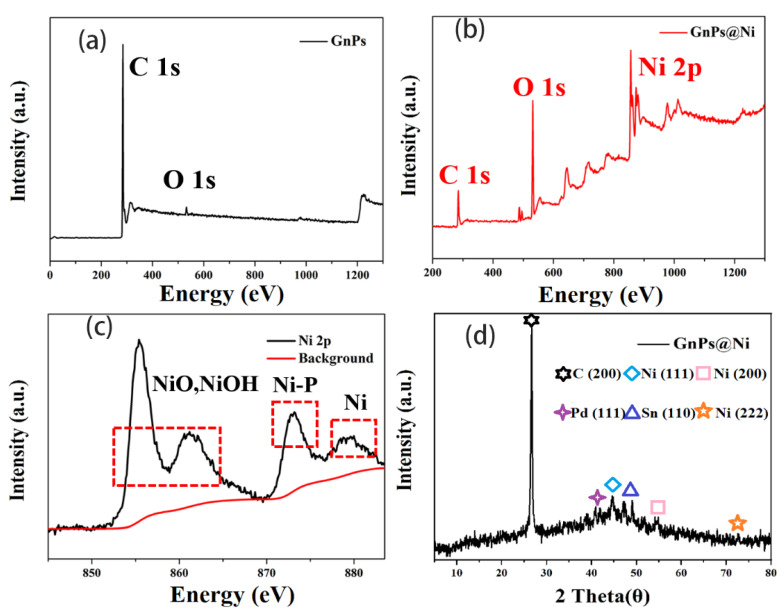
The XPS wide-scan spectrum of GnPs (**a**) and Gnps@Ni (**b**); XPS spectra of Ni 2p in the GnPs@Ni (**c**); the XRD pattern of Gnps@Ni (**d**).

Herein, XPS and XRD spectrum were performed to investigate chemical composition of the modified graphite nanoplatelets after electroless nickel plating process. As shown in Figure 2a–c, the peaks at 281.8 eV and 535.4 eV attributed to C 1s and O 1s, respectively. After treatment, the appearance of new characteristic peaks of Ni 2p in GnPs@Ni at binding energies of 855.3 eV preliminarily proves that nickel particles were deposited on the surface of graphite nanoplatelets successfully. The elemental scan of Ni 2p 4/3 and Ni 2p 5/3 core level peaks of the functional filler being centered at 855.4 eV and 873.1 eV can be observed, respectively. It is noteworthy that the Ni 2p peak had two oxidative satellite peaks, which were derived from trivalent nickel of Ni(OH)_3_ in the coating and finally transformed into NiOOH [25]. Therefore, there are more than one kind of Ni-containing compounds on the surface of graphite nanoplates: Ni, Ni-P alloy, Ni oxides and hydroxides, which further explained the enhancement in intensity of O 1s peak in Figure 2b. In order to further determine the crystal structure of Ni particles and the related reaction process involved, the XRD was carried out. Figure 2d illustrated that aside from the characteristic peaks of GnPs (26.3°), the peaks at 44.8° and 52.1° corresponded to the Ni (111) and Ni (200) crystal face, respectively. Moreover, the additional diffraction peaks were located at 2θ = 40.6° and 51.6°, corresponding to Bragg diffraction peaks of Pd (111) and Sn (110) planes [26]. It proved that Pd and Sn nanoparticles appeared on the surface of the prepared GnPs@Ni, indicating that the Sn metal particles were not completely wrapped by Pd particles. During the whole process, as autocatalytic active centers, most of the Pd particles were coated by Ni nanoclusters; meanwhile, the catalytic ability of Sn was much lower than that of Pd. Therefore, it cannot work as a growth center to participate in the chemical reaction to generate Ni nanoparticles [25].

### 3.2. Morphology of GnPs@Ni-MWCNTs Hybrid Filler

**Figure 3:**
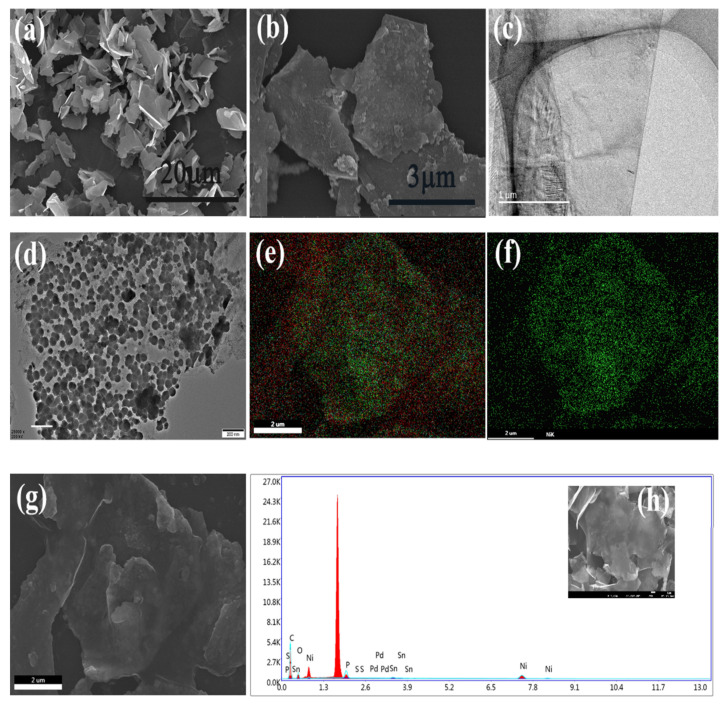
The SEM images of GnPs (**a**) and GnPs@Ni (**b**); TEM images of GnPs (**c**) and GnPs@Ni (**d**); the background and mapping of GnPs@Ni (**e**–**g**); the EDX analysis of GnPs (**h**).

To visualize the Ni-plated graphite nanoplatelets morphology, scanning electron microscopy (SEM) and transmission electron microscopy (TEM) were employed. As demonstrated in Figure 3a,c, it is clear that the graphite nanosheets had a folded and slightly wrinkled morphology composed of a small amount of single-layer graphene. After chemical deposition, many spherical nickel nanoclusters were observed to have appeared on the surface of GnPs, but the particle size was slightly uneven. In addition, it could be seen from TEM that Ni nanoparticles were distributed uniformly, and there was no obvious agglomeration on the surface of GnPs. The distribution of nickel loaded on graphite nanoplatelets was observed to in Figure 3e–g via EDX analysis, and the green part represents the distribution range of nickel elements, thus it is observed that the distribution of nickel in the surface of GnPs@Ni was uniform, which is consistent with the observed phenomena in SEM and TEM. In addition, Figure 3h illustrates that GnPs surface mainly contained C, O, P, Ni, Pd and Sn, accounting for 55.45, 21.25, 10, 12.83, 0.23 and 0.24 wt%.

### 3.3. Thermal Conductivity of GnPs@Ni/PPSU Composites

**Figure 4:**
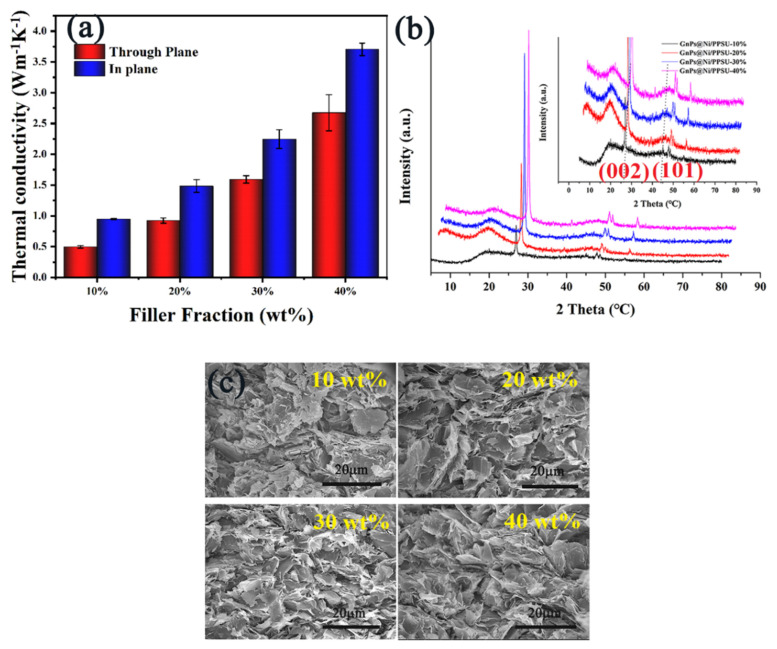
The in-plane and through plane thermal conductivity (**a**), along with XRD spectrum (**b**) of the GnPs@Ni/PPSU composites; the SEM images of the developed composites at different filler loading (**c**).

An upward trend in the TC value of the GnPs@Ni/PPSU composites was seen with the filler content increasing in Figure 4.

For example, in-plane and through-plane λ value of the developed composites can reach up to 2.8 and 3.7 Wm^−1^K^−1^ at 40 wt% filler loading, respectively, indicating the thermal conductivity enhancement (TCE) of modified composites were 10 and 14 times higher than that of pure PPSU matrix. Additionally, it is notable that conduction of heat currents showed obviously anisotropic inside the composites materials, which is mainly caused by the selective arrangement of thermally conducting fillers in the PPSU matrix. Herein, X-ray diffraction (XRD) patterns of the prepared GnPs@Ni/PPSU composites materials containing different amounts of GnPs@Ni was utilized to analyze the orientation degree of the layered fillers in the PPSU matrix qualitatively and roughly. As seen in Figure 3b, there are mainly three diffraction peaks at 2θ = 26.5°, 45.6° and 54.7°, attributed to the (002), (101) and (004) crystallographic planes of GnPs@Ni functional fillers, respectively. The orientation degree of GnPs@Ni can be obtained by the ratio of diffraction peak intensity between (002) and (101) crystal surface (I (002))/(I (101)). The research shows that the filler is easier to be oriented in the horizontal direction under the influence of gravity [27,28]. When the amount of filler increases, the ratios were 2.5, 4.3, 6.9 to 7.2, respectively, which indicates that the layered GnPs@Ni is mainly distributed along the (002) crystal surface [29,30]. Furthermore, according to the SEM spectra, it was found that with the increase of filler content in the GnPs@Ni/PPSU composites, multilayer graphene nanoflakes tended to be distributed along horizontal direction, which was consistent with the above XRD spectrum. Due to the large aspect ratio and good flexibility, layered shaped fillers such as graphene, BN and Mxene, tend to selectively locate in the horizontal direction of the composites during the hot-pressing process under the effect of gravity [31,32]. The intrinsic thermal conductivity of GnPs in the vertical direction is rather low (about 6 Wm^−1^K^−1^).

**Figure 5:**
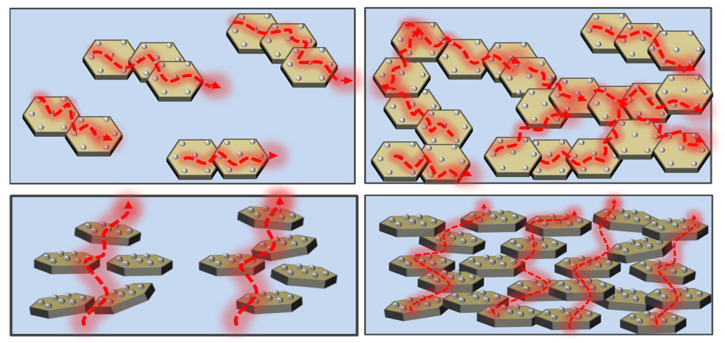
Schematic diagram of ideal heat conduction mechanism of composite materials.

As illustrated in Figure 5, the PPSU matrix acts as heat flow barriers between the fillers, resulting in serious phonons scattering in the vertical direction of composites. On the contrary, in the horizontal direction is the adjacent bridged layered filler, which is beneficial to construct consecutive thermally conductive networks. According to the thermally conductive path theory, the heat currents tend to transfer along the pathways with low interfacial thermal resistance in the polymeric composites, leading to the heat currents transmitting much faster along the horizontal direction. Hence, the thermal conduction presented obvious anisotropy in the composites on the macro level.

**Figure 6:**
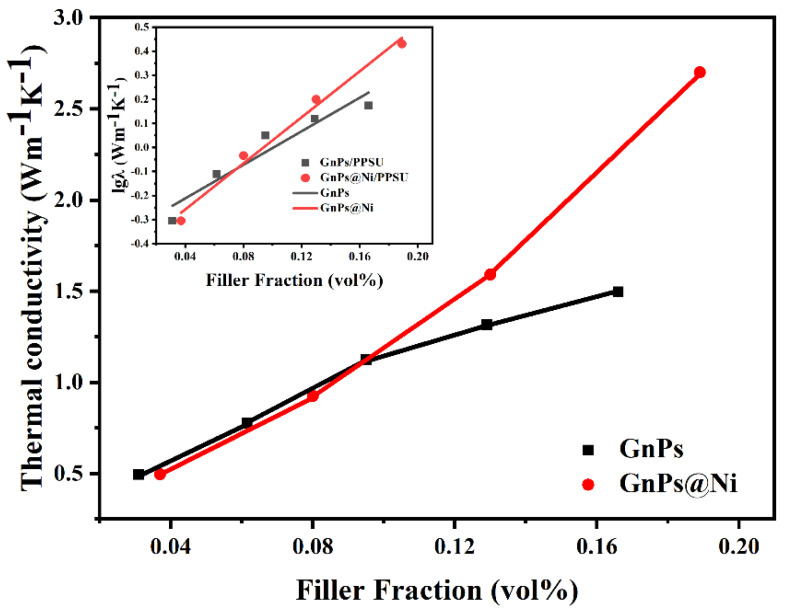
The fitted curve of TC value of the GnPs/PPSU and GnPs@Ni/PPSU composites based on Agari model.

Figure 6 comparatively shows the λ value of GnPs@Ni/PPSU and GnPs/PPSU, and the Agari model is utilized to analyze the effects of Ni layer on the thermal conductivity enhancement. When the filling content was low, both GnPS@Ni/PPSU and GnPs/PPSU composites exhibited similar thermal conductivity. Nevertheless, the λ value of the composite is significantly higher than that of GnPs/PPSU when the filler amount is beyond 10 vol%. Subsequently, the Agari model was used to weigh up the capability of two functional fillers of forming heat conductive networks in the nanocomposites.

$$\log\lambda_{c}=V_{f}C_{2}{\;\log\lambda }_{f}+\left(1-V_{f}\right)\log\left(C_{1}\lambda_{m}\right)$$

where V_f_, λ_f_, λ_m_, λ_c_ represented volume percentage of fillers, thermal conductivity of fillers, matrix and developed composite, respectively, with a view to assess those impact for dispersion of conductive fillers for thermal management. The variables for C_1_ and C_2_ are presented. C_1_ is an element reflecting the impact for crystallinity and crystal size of matrix on the thermal conduction capacity of composite. When C_1_ < 1, the filler will affect the structure of the matrix, thus affecting the thermal conductivity of the material. On the contrary, when C_1_ > 1, it will not affect the thermal conductivity. C_1_ will approach 1 under ideal condition, which proposes that secondary structure of polymer was slightly influenced by the addition of fillers, especially for the thermal conductivity. C_2_ represents the contact tightness of filler particles, that is, the ability of forming a heat conduction network. In the specification, C_2_ is 0~1, but in practical application, the greater the C_2_ value, the composites, therefore the stronger the ability to form a heat conduction network. As shown in Table 1, first, the result shows that the incorporation of GnPs@Ni and GnPs has no influence on the secondary structure of PPSU. Instead, compared to GnPs/PPSU, C_2_ was improved obviously in the GnPs@Ni/PPSU, indicating the ability of GnPs to form thermal conductive pathways was effectively enhanced after electroless plating [33,34]. In contrast to covalent modification, surface modification by chemical deposition will not destroy the crystal structure and physical properties of thermal conductive fillers. This may be because the introduction of the metal layer can enlarge the contact area between the fillers, leading to the increment in the amount of the heat conductive pathways.

### 3.4. EMI Shielding Performance of GnPs@Ni-MWCNTs/PPSU Composites

**Figure 7:**
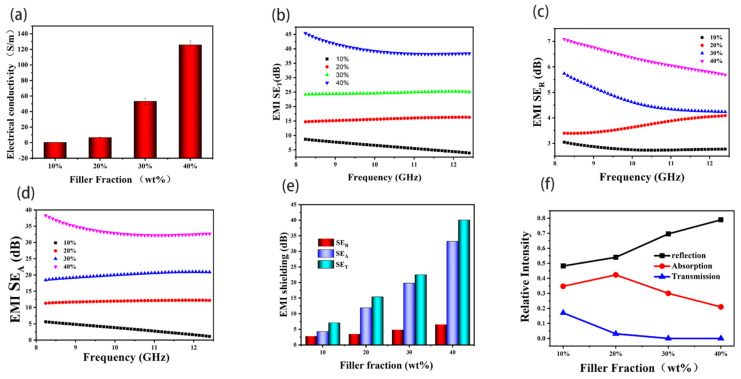
The electrical conductivity (**a**), EMI SET (**b**), EMI SER (**c**), EMI SEA (**d**) of GnPs@Ni/PPSU composites; EMI SE of the composites as a function of volume fraction at 9 GHz (**e**); reflection coefficient (R), absorption coefficient (A), and transmission coefficient (T) at 9 GHz of the developed composites (**f**). The thickness of all samples was 2 mm.

Based on the plane electromagnetic wave conduction theory advanced by Schelkunoff, perfect conductive network structures are one of prerequisites to obtain high EC value and electromagnetic shielding effectiveness [35,36]. The EC value of the GnPs@Ni/PPSU composites is presented in Figure 7a. The EC rose sharply with the increase of filler amount, verifying that successive conductive path has been established. The EC value was achieved to 128.3 S/m at 40 wt% filler loading, which is nearly 15 orders of magnitude higher than that of pure PPSU (around 10–13). The EMI shielding effectiveness of the prepared PPSU composites on frequency ranging from 8.4 to 12.8 GHz can be seen in Figure 7b–d. With the amount of functional particles GnPs@Ni increasing, the total shielding effectiveness (SET) and absorption efficiency (SEA) were significantly boosted, contrarily, the reflection efficiency (SER) showed a slight improvement from around an average of 2.5 to 6.8 dB. The maximum SET, SEA and SER reached 7.1, 38.3 and 42.9 dB, respectively, at the 40 wt% filler loading, which far exceeds the commercial demand (20 dB). The transmission (T), absorption (A) and reflection efficiency (R) of the composites are calculated according to the electromagnetic shielding formula. Consequently, the reflectivity increases from 0.48 to 0.79 with the increase of filler content as shown in 3.7 (d) [25,37]. Therefore, it can be inferred that the modified GnPs@Ni/PPSU composite was high-performance reflection-dominated electromagnetic shielding material [38]. The likely mechanism could be explained as follows. When the electromagnetic wave reaches the surface of the composite, since GnPs@Ni/PPSU composites have very high conductivity, the impedance mismatch of the interphase between air and composites gives rise to large amounts of electromagnetic waves to be reflected. As mentioned, reflectivity holds a large proportion of the whole shielding effectiveness. Meanwhile, owing to GnPs@Ni fillers having weak magnetism, once the residual electromagnetic waves enter interior of materials, they will be absorbed and exhausted, effectively achieving the purpose of attenuating the electromagnetic wave. Therefore, it is difficult to penetrate the material [18,25,35].

### 3.5. Thermal Analysis of the GnPs@Ni-MWCNTs/PPSU Composites

**Figure 8:**
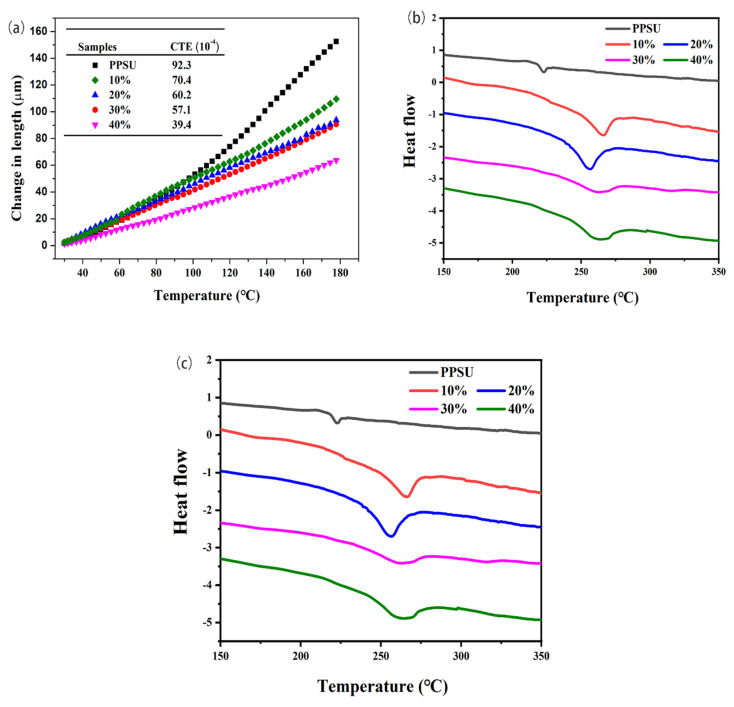
Coefficient of thermal expansion of pure PPSU resin and the GnPs@Ni-MWCNTs/PPSU nanocomposites (**a**); thermal properties of PPSU and the GnPs@Ni-MWCNTs/PPSU composites: DSC (**b**) and TGA (**c**) thermograms.

The coefficient of thermal expansion (CTE) of materials reflects the parameter of dimensional stability of materials as temperature changes. When aerospace materials and electronic devices are used in extreme conditions, such as large temperature difference between day and night, different components may have uneven volume expansion, resulting in huge internal stress and low accuracy [12]. Reducing the CTE coefficient of materials is helpful to solve the problem of size mismatch between different components in precision components after heating. Figure 8a comparatively shows the coefficient of thermal expansion of pure PPSU and the prepared composites containing different amounts of GnPs@Ni. The CTE value of pure PPSU resin was 92.3 × 10^−6^ K^−1^. It is noticed that the CTE value was significantly reduced with the fillers loading ranging from 10 wt% to 40 wt%. When the filling amount is 40 wt%, the thermal expansion coefficient was 39.4 × 10^−6^ K^−1^, decreasing by 57.3% compared to that of pure PPSU. The reasons for the decrease of the coefficient of thermal expansion are as follows. The results show that the filler particles can effectively restrain the chain movement of the polymer at high temperature; GnPs@Ni, as an inorganic filler, has a polar linear coefficient of thermal expansion, so the total volume change rate of the composite decreases when heated [32]. The glass transition reflects the inherent properties of amorphous polymers and shows the long chain movement of polymers on the macro level. The addition of fillers will affect the chain segment movement of polymer molecules and the melting behavior of PPSU resin. Therefore, it has a great influence on the temperature of material and the processing and production. In Figure 8b, the differential scanning calorimetry (DSC) curves revealed that the glass transition temperatures (T_g_) of fabricated composites were generally elevated with the increasing content of GnPs@Ni. It can be seen that in the GnPs@Ni/PPSU-10 wt% composite it has increased up to 264.7 °C with respect to PPSU (221.9 °C). The phenomenon was caused by the fact that, with the addition of GnPs increasing, the packing particles could effectively restrain the chain movement of polymer via physical constraints, so the movement of polymer chain segment required more energy.

For the sake of exploring the thermostability of the prepared composites, thermogravimetry (TG) test was carried out and the results are shown in the Figure 8c. All the composites mainly presented a similar thermal weightlessness platform, corresponding to the decomposition of the main chain of PPSU. All of the fabricated composites exhibited outstanding thermostability, with high thermal decomposition temperatures of more than 500 °C (T5%). In addition, the thermal stability of composite material is equivalent to that of pure resin, and the 10 wt% thermal decomposition temperature is approximately beyond 524 °C, which indicates that introduction of GnPs@Ni enhanced the thermal decomposition stability of the material. Importantly, it also showed to slow the decomposition process and higher residual char. Especially, the composite residue will be nearly 75% at 40 wt% filler loading. The obvious enhancement in thermal stability is attributed to the high thermal conductivity of graphene and the good interface bonding between the fillers and the matrix [39]. As a consequence, the increase in heat transfer capacity between the chain segments and the PPSU could reduce the heat storage in the composite [40]. In addition, the improvement of T5% and T10% is the result of the strong shielding effect of GnPs, which significantly delayed the escape of the degradation products of PPSU, and the thermal stability of the composite was improved [32,41].

## 4. Conclusions

The surface of graphite nanoplatelets was modified via non-covalent method by electroless plating. Pd particles were used as an autocatalytic active center to promote the formation of Ni-P alloy and Ni particles in electroless nickel plating. The nickel coating was evenly distributed on the surface of nano graphite. The GnPs@Ni/PPSU composites showed excellent thermal conductivity. The in-plane and through-plane thermal conductivity can reach 2.7 Wm^−1^K^−1^ and 3.7 Wm^−1^K^−1^, respectively, at 40 wt% GnPs@Ni filler loading, which is 10 and 14 times higher than that of pure PPSU. The XRD diffraction patterns and SEM images illustrated that layered GnPs@Ni tended to distribute along the horizontal direction of polymer matrix. Furthermore, the composite material has good conductivity of 128 S/m and electromagnetic interference effectiveness, and it is an electromagnetic shielding material based on reflection. The maximum EMI SE of the material can reach 42.9 dB when the filling amount is 40 wt%. In addition, the composites had lower CTE, higher T_g_ and superior thermal decomposition ability. Overall, the materials had potential applications in the fields of thermal management and electromagnetic shielding interference at high temperatures.

## Figures and Tables

**Table 1:** The comparison of C_1_ and C_2_ of GnPs/PPSU and GnPs@Ni/PPSU.

	C_1_	C_2_
GnPs/PPSU	1.76	1.24
GnPs@Ni/PPSU	1.42	1.4

## Data Availability

The data presented in this study are available on request from the corresponding author.
